# Expressions of miR-302a, miR-105, and miR-888 Play Critical Roles in Pathogenesis, Radiotherapy, and Prognosis on Rectal Cancer Patients: A Study From Rectal Cancer Patients in a Swedish Rectal Cancer Trial of Preoperative Radiotherapy to Big Database Analyses

**DOI:** 10.3389/fonc.2020.567042

**Published:** 2020-10-06

**Authors:** Wen-Jian Meng, Surajit Pathak, Xueli Zhang, Gunnar Adell, Ingvar Jarlsfelt, Birgitta Holmlund, Zi-Qiang Wang, Alexander S. Zhang, Hong Zhang, Zong-Guang Zhou, Xiao-Feng Sun

**Affiliations:** ^1^Department of Oncology and Department of Biomedical and Clinical Sciences, University of Linköping, Linköping, Sweden; ^2^Department of Gastrointestinal Surgery, West China Hospital, Sichuan University, Chengdu, China; ^3^Chettinad Hospital & Research Institute, Chettinad Academy of Research and Education, Kelambakkam, India; ^4^School of Medicine, Institute of Medical Sciences, Örebro University, Örebro, Sweden; ^5^County Council of Östergötland, University of Linköping, Linköping, Sweden; ^6^Department of Pathology, Ryhov Hospital, Jönköping, Sweden; ^7^Department of Oncology-Pathology, Karolinska Institute, Stockholm, Sweden; ^8^State Key Laboratory of Biotherapy and Cancer Center, Institute of Digestive Surgery, West China Hospital, Sichuan University, Chengdu, China

**Keywords:** miRNAs, pathogenesis, radiotherapy, prognosis, rectal cancer

## Abstract

Differential expressions and functions of various micoRNAs (miRNAs) have been intensively studied in both colon and rectal cancers. However, the importance of miRNAs on radiotherapy (RT) response and clinical outcome in rectal cancer patients remains unclear. In this study, we used real-time polymerase chain reaction to examine the expressions of miR-302a, miR-105, and miR-888 in normal mucosa and cancer tissue from rectal cancer patients with and without preoperative RT. The biological function of miR-302a, miR-105, and miR-888 expression was further analyzed and identified through the public databases: TCGA (The Cancer Genome Atlas) and GEPIA (Gene Expression Profiling Interactive Analysis). The results showed that the expression of miR-105 in rectal cancer was higher than that in normal mucosa in RT (*P* = 0.042) and non-RT patients (*P* = 0.003) and was associated with mucinous histological type (*P* = 0.004), COX-2 (*P* = 0.042), and p73 expression (*P* = 0.030). The expression of miR-302a was shown more frequently in cancers with necrosis (*P* = 0.033) and with WRAP53 expression (*P* = 0.015), whereas miR-888 expression occurred more frequently in tumors with protein the expression of survivin (*P* = 0.015), AEG-1 (astrocyte elevated gene-1) (*P* = 0.003), and SATB1 (special AT-rich sequence binding protein 1) (*P* = 0.036). Moreover, TargetScan also predicted AEG-1 and SATB1 as putative targets for miR-888. The miRNA–gene network analysis showed that ABI2 was associated with all the three miRNAs, with lower expression and good diagnostic value in rectal cancers. The TCGA database demonstrated the association of miR-105 expression with high carcinoembryonic antigen level (*P* = 0.048). RT reduced the expressions of miR-302a, miR-105, and miR-888. Prognostic analysis showed that miR-888 expression was independently associated with worse survival of patients without RT [overall survival, *P* = 0.001; disease-free survival, *P* = 0.009]. Analysis of biological function revealed that the protein serine/threonine kinase activity and PI3K-AKT signaling pathway were the most significantly enriched functions and pathways, respectively. Our findings suggest that miR-105 is involved in rectal cancer pathogenesis and miR-888 is associated with prognosis. MiR-302a, miR-105, and miR-888 have potential influence on the pathogenesis, RT, and prognosis of rectal cancer.

## Introduction

Colorectal cancer (CRC) includes both colon and rectal cancers and is the third most common cancer worldwide and the second leading cause of cancer deaths in many parts of the Western world (1). Because of the differences in embryological origin, anatomy, and biological and clinical features, such as metastatic patterns, colon, and rectal cancers should require separate strategies concerning cancer therapies. Adjuvant chemotherapy in stage III and possibly high-risk stage II colon cancer is associated with improved survival (2). Preoperative short- or long-course radiotherapy (RT) for stage II or III rectal cancer is the standard course of treatment. Total mesorectal excision is the cornerstone of rectal cancer management. Although the multimodal therapies including a preoperative radiochemotherapy have been proposed for patients with locally advanced rectal cancer based on TNM staging according to National Comprehensive Cancer Network guidelines, the response to RT and prognosis vary a lot among patients who have the same TNM stages. Therefore, it is urgent to discover novel and better biomarkers as clinical indicators to improve RT response and eventually survival in rectal cancer patients.

MicroRNAs (miRNAs) are small non-coding RNAs (18–22 nt in length) that regulate the expression of target genes by interfering at posttranscriptional level by degrading target mRNAs and/or inhibiting their translation (3). Increasing amounts of evidence show that miRNAs play a crucial role in almost all cellular biological processes including metabolism, differentiation, survival, and apoptosis. The discovery of critical functions of miRNAs has opened new avenues for diagnosis, prediction of treatment response, and prognosis for many malignancies including CRC (4). PI3K/AKT signaling miR-105 expression has been altered in different types of cancers. Loss of miR-105 expression was associated with poor prognosis in hepatocellular carcinoma (HCC) (5, 6), glioma (7, 8), and non–small cell lung cancer (9), which indicates that miR-105 may function as a potential tumor suppressor in these malignancies. On the contrary, upregulated expression of miR-105 was associated with poor prognosis in breast cancer patients (10). Moreover, overexpression of miR-105 was associated with CRC with aggressive phenotype (11). However, the specific clinical values of miR-105 in CRC remain to be further elucidated. Aberrant expression of miR-888 has recently been reported in several types of cancers, prominently in urogenital malignancies. MiR-888 has been shown to be dysregulated in chromophobe renal cell carcinomas (12). It has been found that miR-888 is involved in the promotion of prostate cancer progression (13), and a recent study identified miR-888 cluster as a novel cancer network in prostate cancer (14). In addition, an infrequent somatic mutation mapping within the miR-888 hairpin is identified in a primary epithelial ovarian tumor (15). MiR-888 is one of the most overexpressed small RNAs in endometrial cancers (16, 17). MiR-888 may be involved in breast cancer metastasis (18), and plays a major role in promoting the proliferation and metastatic potential of HCC cells (19). These findings indicate that miR-888 plays an oncogenic role in several cancer types. Although the overexpression of miR-888 has been found in colon cancer tissue as compared with normal mucosa by large-scale miRNA profiling (20), the potential role of miRNA-888 in CRC remains to be further investigated. The aberrant expression of miR-302a has been frequently reported in a variety of cancer types. The downregulation of miR-302a in breast cancer cells plays an important role in regulation of invasion and metastasis of breast cancer (21). The low expression of miR-302a inhibits cell proliferation and invasion and induces cell apoptosis in HCC by directly targeting vascular endothelial growth factor-A (22). The overexpression of miR-302a in prostate cancer cells can induce cell cycle arrest and inhibit cell proliferation *in vitro* and tumor formation *in vivo* (23). Moreover, the lower expression of miR-302 family members is associated with more aggressive cancer progression in skin, cervical, and gastric cancer (24–27). MiR-302a is overexpressed in colon cancer (20), which may inhibit proliferation and invasion of cancer cells by reducing the expression of related proteins through suppressing the MAPK and PI3K/AKT signaling pathways (28, 29). However, the prognostic value of miR-302a in CRC has been not completely clarified.

It is well-known that one single miRNA can simultaneously target multiple genes that are involved in a specific signaling cascade or cellular mechanisms. Therefore, some miRNAs may be regulators of complex radioresistance responses of tumor cells and thus directly exploited to sensitize radioresistant CRC cells to RT. For instance, miR-302a can sensitize the response of breast cancer to RT (30), and miR-302a expression is inversely correlated with those of AKT1 and RAD52 (two critical regulators of radioresistance). To our knowledge, there is no such study that has been specifically conducted to investigate the involvement of miR-302a, miR-105, and miR-888 in RT response of rectal cancers. Therefore, we have taken three steps for selecting miR-302a, miR-105, and miR-888 as the target miRNAs in the present study: (1) by bibliographic retrieval to find their expressions in different malignancies, (2) by studying target miRNAs in different publications based on their important roles in other malignancies, and (3) by finding the data on the prognostic value and RT response in rectal cancer.

This study aimed to examine whether the expressions of miR-302a, miR-105, and miR-888 were associated with other biological factors, RT response, and clinical outcome in a cohort of rectal cancer patients obtained from Swedish Clinical Trial of Preoperative RT. Our specified aims, based on RT or non-RT patients, were to examine the expressions of the three miRNAs in the primary cancers compared with normal rectal mucosa, RT effects on the miRNAs, the relationships of the miRNAs with proliferation- and apoptosis-related factors, and patient survival. We further analyzed these miRNAs in the protein-protein interaction network and their biological functions based on The Cancer Genome Atlas (TCGA) and Gene Expression Profiling Interactive Analysis (GEPIA) database.

## Materials and Methods

### Rectal Cancer Patients and Samples

This study included a cohort of 80 patients with rectal cancer from the Southeast Swedish Health Care region who participated in a randomized Swedish Rectal Cancer Trial of Preoperative RT between 1987 and 1990 (31). Locally curative resection was performed on all patients. The surgery has been described in detail (31). Forty-one patients received tumor resection alone, and 39 received preoperative RT followed by surgical tumor resection. RT was given at a total dose of 25 Gy in five fractions over a median of 8 days (6–14 days) before the surgery. The surgical tumor resection was carried out in a median of 4 days (range, 0–8 days) after RT. Samples were collected from primary rectal cancer (*n* = 80) and distant normal mucosa (*n* = 52). The distant normal mucosa was taken from the proximal or distal margin (4–35 cm from the primary tumor) of the resected rectum, which was histologically free from tumor. Tissue collection and preparation are described in detail in our previous study (32). None of the patients received preoperative or adjuvant chemotherapy. The median follow-up period or the patients was 66.5 months (range, 0–193 months). All patients gave their informed consent for inclusion before they participated in the study. The study was conducted in accordance with the Declaration of Helsinki, and the protocol was approved by the institutional review board of Linköping University, Sweden (Dnr-2012-107-31). There was no statistical difference between the non-RT and RT patients regarding the characteristics of the patients and tumors (*P* > 0.05; Table 1).

**Table 1:** Characteristics of the patients and tumors.

**Characteristics**	***n***	**Non-RT (%)**	**RT (%)**	***P*-value**
**Gender**				
Male	47	23 (48.9)	24 (51.1)	0.621
Female	33	18 (54.5)	15 (45.5)	
**Age (year)**				
≤66	29	14 (48.3)	15 (51.7)	0.688
>66	51	27 (52.9)	24 (47.1)	
**TNM stage**				
I	28	13 (46.4)	15 (53.6)	0.652
II	19	9 (47.4)	10 (52.6)	
III	28	17 (60.7)	11 (39.3)	
IV	5	2 (40)	3 (60)	
**Histological grade**				
Well + moderate	61	33 (54.1)	28 (45.9)	0.361
Poor	19	8 (42.1)	11 (57.9)	
**Surgical type**				
Anterior resection	41	21 (51.2)	20 (48.8)	0.996
Abdominoperineal	39	20 (51.3)	19 (48.7)	
**To anal verge (cm)**				
Mean	70	6.854 ± 3.896	8.128 ± 5.242	0.219

*Non-RT/RT: the rectal cancer patients without/with preoperative radiotherapy*.

### Quantitative Reverse Transcriptase–Polymerase Chain Reaction for miRNA Expression

An experienced pathologist took samples including normal mucosa and primary tumor from surgical specimens. The samples were formalin-fixed and paraffin-embedded, and the tissue blocks were sectioned to produce standard microscopic slides that were stained with hematoxylin and eosin (HE). An experienced pathologist examined all HE slides from different pathological blocks from each patient in order to select our required block/tissue for use. Total RNA was extracted from cancerous and distant normal samples from patients, using mirVana miRNA Isolation Kit (Ambion, Austin, TX, USA) according to the manufacturer's protocol. Concentration and purity of RNA were quantified using Nanodrop ND-1000 Spectrophotometer (Thermo Scientific, Rockford, IL, USA). cDNA was synthesized using gene-specific primers according to the TaqMan MicroRNA Assay protocol (Applied Biosystems). After reverse transcription and quantitative reverse transcriptase–polymerase chain reaction (qRT-PCR), the CT values (CT) were calculated by SDS 2.4.1 software (Applied Biosystems) using the manual threshold settings (threshold = 0.2). RNU6b (assay no. 001006; Applied Biosystems) was selected as a reference gene. The fold change in miRNA expression was calculated using the 2^−ΔΔCt^ method (33). The detailed protocol for qRT-PCR and data analysis was described in Supplementary Materials. Finally, TargetScan 3.0 was then used to predict gene targets of these miRNAs.

### Tissue Array and Immunohistochemistry for the Expression of Biological Factors

In order to understand the possible pathway of miRNAs in carcinogenesis, RT response, and eventually patient survival, we examined the relationships of the miRNAs with biological factors associated with apoptosis, carcinogenesis, and RT response. These factors were studied on the sections of the blocks (fixation described above) from the same patients at our laboratory. The data for p73 (*n* = 74) (32), p130 (*n* = 71) (34), phosphatase of regenerating liver-3 (PRL-3, *n* = 73) (35), endosialin (TEM1, *n* = 75) (36), survivin (*n* = 47) (37), peroxisome proliferator-activated receptor δ (PPAR-δ, *n* = 67) (38), WRAP53 (*n* = 67) (39), astrocyte elevated gene-1 (AEG-1) (*n* = 70) (40), COX-2 (*n* = 77) (41), and special AT-rich sequence binding protein 1 (SATB1, *n* = 68) (42) of primary rectal cancers determined by immunohistochemistry were taken from previous studies conducted with the same cases used in the present study at our laboratory. Apoptosis (*n* = 71) was detected by TUNEL (terminal deoxynucleotidyl transferase-mediated deoxyuridine triphosphate-biotin nick end labeling) assay (43). The percentage of apoptotic cancer cells was determined by counting ~1,000 tumor cells. Cases were considered as negative if apoptotic cells constituted fewer than 5% of tumor cells. The detailed protocol is described in Supplementary Materials.

### miRNA–Gene Network Construction

The miRNet database provided the miRNA–gene interaction information, and Cytoscape software was used to visualize the networks.

### Biological Functional Analysis

Gene Ontology (GO) annotation and Kyoto Encyclopedia of Genes and Genomes (KEGG) pathway enrichment analysis conducted by “clusterProfiler” package on R language were used to explore the biological function for miRNA-related genes on pathway level.

ABI2 expression was compared between rectal cancer tissues (*n* = 92) and normal controls (*n* = 318) based on the GEPIA database. Further, the diagnostic values of ABI2 expression were analyzed in 167 rectal cancer patients and 10 normal controls based on the TCGA database. Finally, the prognostic value of ABI2 expression in 92 rectal cancer patients was determined by survival analysis.

### TCGA Data Analysis

The data of miR-105, miR-302a, and miR-888 expression for 170 rectal cancer and three paired normal rectal specimens were downloaded from TCGA data portal (National Cancer Institute and National Human Genome Research Institute, accessed November 1, 2017). The data collection process was in compliance with all laws and regulations. Clinical characteristics are summarized in Table 2, except one case, which was excluded because of the nature of treatment being neoadjuvant. Then, clinical significance and prognostic value of three miRNAs for rectal cancer based on TCGA were also investigated.

**Table 2:** TCGA rectal cancer clinical characteristics.

**Characteristics**	**Category**	**Number of specimens**	**Percentage**
miR-105 expression	Zero	55	32.4%
	Non-zero	105	61.7%
	NA	10	5.9%
miR-302a expression	Zero	155	91.2%
	Non-zero	5	2.9%
	NA	10	5.9%
miR-888 expression	Zero	153	90%
	Non-zero	7	4.1%
	NA	10	5.9%
Gender	Female	78	45.9%
	Male	92	54.1%
TNM stage	I	33	19.4%
	II	51	30.0%
	III	52	30.6%
	IV	25	14.7%
	NA	9	5.3%
Tumor histology	Adenocarcinoma	151	88.8%
	Mucinous Adenocarcinoma	13	7.6%
	NA	6	3.6%
Lymphatic invasion	Yes	68	40.0%
	No	84	49.4%
	NA	18	10.6%
Venous invasion	Yes	38	22.4%
	No	109	64.1%
	NA	23	13.5%
Preoperative CEA (ng/ml)	<3.4	63	37.1%
	≥3.4	51	30%
	NA	56	32.9%
History of neoadjuvant treatment	Yes	169	99.4%
	No	1	0.6%

*NA, not available*.

### Statistical Analysis

Statistical differences in miRNA expression between cancer and distant normal samples were evaluated by the two-tailed non-parametric Wilcoxon test by GraphPad Prism 5.0 (GraphPad Software Inc., San Diego, CA, USA). The relationships of miRNA levels with clinicopathological parameters or biological variables were evaluated by using the Mann-Whitney *U*-test for two groups and the Kruskal–Wallis test for more than two groups. Survival curves were generated according to the Kaplan–Meier method and compared by the log-rank test. Multivariate analyses were performed with the Cox proportional hazards model. *P* < 0.05 was considered significant. These calculations were performed with Statistica version 10.0 (StatSoft Inc.).

## Results

### The Expression of miR-302a, miR-105, and miR-888

**Figure 1:**
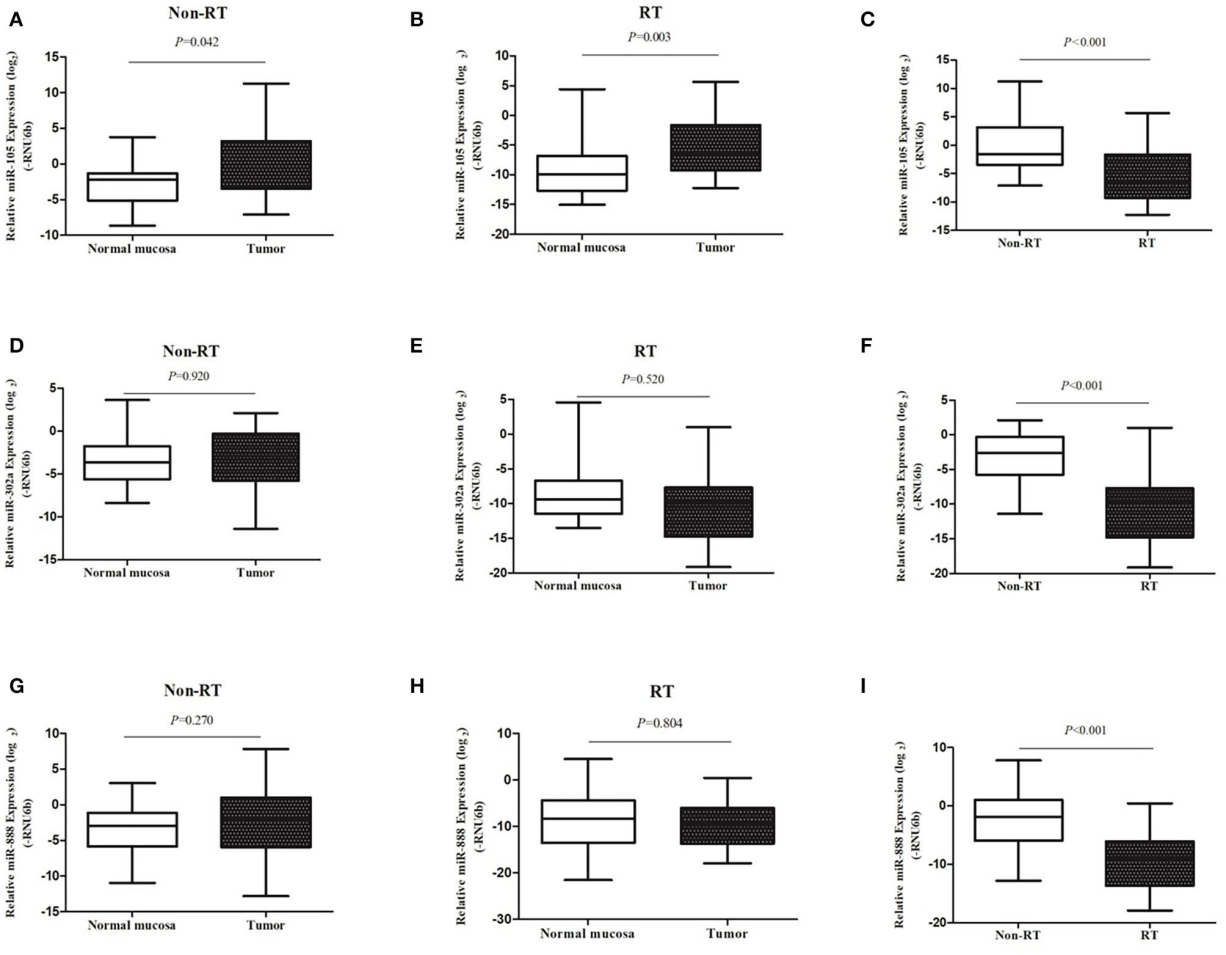
miRNA expression was examined with qRT-PCR. The expression levels of miR-105 **(A–C)**, miR-302a **(D–F)**, and miR-888 **(G–I)** in rectal cancers as compared with the normal mucosa in patients without and with RT were represented in the box–whisker plots on a log_2_ scale.

The expression of miR-302a, miR-105, and miR-888 was examined in 80 primary rectal cancers and 56 normal rectal samples normalized to RNU6b. Expression of each miRNA in cancers with normal samples was compared in non-RT group and RT group. As shown in Figure 1, the expression of miR-105 was significantly increased in cancers compared with normal samples in non-RT cases (median, 0.041 vs. 0.027, *P* = 0.042). A similar phenomenon was observed in RT cases; i.e., miR-105 expression was significantly increased in cancers compared with normal samples (median, 0.116 vs. 0.012, *P* = 0.003). There were no differences in the expressions of miR-302a and miR-888 between cancers and normal samples regardless of RT.

We then compared each miRNA expression of cancers between non-RT and RT group. Compared with the non-RT cases, the expression of miR-105 in RT cases was significantly decreased in cancers (median, 0.334 vs. 0.010, *P* < 0.001) when the normalization of the expression of miR-105 of normal samples was 1. However, compared with the non-RT cases, the expression of miR-302a and miR-888 in RT cases were significantly decreased in cancers (median, 0.169 vs. 0.001, *P* < 0.001; median, 0.270 vs. 0.002, *P* < 0.001; Figure 1).

### miRNA Expression in Relation to Clinicopathological and Biological Features

In non-RT cases, the high expression of miR-105 was significantly more common in mucinous cancers than non-mucinous histological type (*P* = 0.004, Table 3). The high expression of miR-105 was markedly associated with strong COX-2 and positive p73 expression (*P* = 0.030, Table 4). Likewise, miR-302a expression was more frequent in the cancers with necrosis (*P* = 0.033, Table 3) and strong WRAP53 expression (*P* = 0.015, Table 4). The cancer patients with the high expression of miR-888 were older than those with low miR-888 expression (*P* = 0.038, Table 3). The high expression of miR-888 also occurred more frequently in the cancers with strong survivin expression (*P* = 0.015) and the weak expression of AEG-1 (*P* = 0.003) and SATB1 (*P* = 0.036, Table 4). The TargetScan miRNA target prediction algorithm identified little overlap between the targets identified with the database and our experimental results except two targets (AEG-1 and SATB1) for miRNA-888 (Supplementary Materials). There were no significant associations between the expressions of miR-302a, miR-105, miR-888, and gender or TNM stage.

**Table 3:** Correlation between miR-105, miR-302a, and miR-888 expression and clinicopathological features in rectal patients without and with radiotherapy (RT).

	***n***	**Non-RT**			***n***	**RT**		
		**miR-105**	**miR-302a**	**miR-888**		**miR-105**	**miR-302a**	**miR-888**
**Tumor vs. mucosa**								
Normal mucosa	27	0.027 (0.004–0.050)	2.472 (0.826–9.098)	1.007 (0.136–3.494)	25	0.012 (0.002–0.097)	0.310 (0.082–1.836)	1.212 (0.034–19.616)
Rectal tumor	41	0.041 (0.111–1.113)	5.184 (0.560–25.341)	2.090 (0.127–15.516)	39	0.116 (0.018–3.517)	0.284 (0.008–1.126)	0.740 (0.031–6.004)
*P*-value		0.042	0.920	0.270		0.003	0.520	0.804
**Gender**								
Male	23	0.014 (0.006–1.182)	0.287 (0.065–3.606)	2.088 (0.071–6.506)	24	0.055 (0.015–1.335)	0.163 (0.004–2.712)	0.341 (0.007–4.144)
Female	18	0.045 (0.014–1.511)	0.540 (0.427–1.646)	4.970 (0.606–58.337)	15	1.838 (0.107–4.019)	0.402 (0.184–1.126)	1.355 (0.184–7.725)
*P*-value		0.415	0.713	0.270		0.044	0.338	0.138
**Age (year)**								
≤66	14	0.038 (0.013–0.967)	0.424 (0.026–2.023)	0.799 (0.002–6.063)	15	0.364 (0.042–4.019)	0.145 (0.004–0.942)	0.740 (0.008–10.531)
>66	27	0.042 (0.006–1.182)	0.518 (0.104–3.610)	3.780 (0.793–51.700)	24	0.083 (0.015–1.833)	0.353 (0.071–5.028)	0.854 (0.127–3.579)
*P*-value		0.869	0.257	0.038		0.283	0.296	0.989
**TNM stage**								
I	13	0.047 (0.005–0.963)	0.128 (0.023–2.301)	2.090 (0.012–17.507)	15	0.227 (0.010–1.817)	0.352 (0.027–0.942)	0.375 (0.026–6.214)
II	9	0.065 (0.009–0.807)	0.117 (0.036–2.679)	1.180 (0.022–219.952)	10	0.556 (0.043–8.825)	0.202 (0.042–9.790)	1.046 (0.089–8.426)
III	17	0.034 (0.013–1.958)	0.561 (0.200–2.533)	1.790 (0.420–20.122)	11	0.084 (0.032–4.019)	0.284 (0.003–3.302)	1.759 (0.006–5.692)
IV	2	0.014 (–)	0.691 (–)	5.420 (–)	3	1.447 (0.014–11.258)	0.422 (0.145–1.126)	1.126 (0.318–70.216)
*P*-value		0.875	0.375	0.417		0.735	0.379	0.797
**Histological grade**								
well + moderate	33	0.033 (0.011–0.3821)	0.291 (0.045–2.534)	2.818 (0.057–17.499)	28	0.236 (0.033–3.097)	0.318 (0.045–1.080)	1.240 (0.032–6.080)
Poor	8	4.343 (0.025–55.403)	0.571 (0.345–2.952)	1.291 (0.353–15.001)	11	0.084 (0.010–3.753)	0.062 (0.002–3.302)	0.375 (0.008–6.000)
*P*-value		0.058	0.511	0.669		0.233	0.396	0.548
**Necrosis**								
Negative	23	0.042 (0.011–0.713)	0.287 (0.041–1.339)	2.090 (0.015–22.217)	21	0.244 (0.017–3.885)	0.284 (0.006–1.036)	1.355 (0.321–6.109)
Positive	14	0.062 (0.013–1.401)	0.838 (0.418–4.777)	2.847 (0.149–17.811)	17	0.107 (0.017–1.830)	0.220 (0.034–3.575)	0.182 (0.009–4.924)
*P*-value		0.506	0.033	0.722		0.816	0.642	0.170
**Fibrosis**								
Weak	16	0.050 (0.007–0.617)	1.115 (0.052–3.942)	1.700 (0.354–6.292)	21	0.364 (0.027–6.250)	0.100 (0.004–0.794)	1.126 (0.073–5.845)
Moderate	10	0.014 (0.006–0.293)	0.417 (0.089–0.738)	4.270 (0.003–42.731)	11	0.058 (0.016–3.750)	0.736 (0.194–14.300)	0.318 (0.006–4.160)
Strong	11	0.432 (0.033–1.837)	0.582 (0.043–3.838)	1.200 (0.009–83.876)	6	0.075 (0.009–0.630)	0.202 (0.003–2.236)	1.286 (0.139–14.190)
*P*-value		0.189	0.612	0.983		0.364	0.056	0.640
**Mucinous**								
Negative	29	0.015 (0.009–0.211)	0.582 (0.047–3.722)	1.788 (0.043–7.249)	31	0.107 (0.016–3.753)	0.194 (0.008–0.848)	0.375 (0.031–5.690)
Positive	8	1.113 (0.175–19.958)	0.426 (0.108–3.001)	61.981 (0.231–784.614)	7	0.227 (0.018–3.520)	0.352 (0.002–8.090)	1.130 (0.012–6.210)
*P*-value		0.004	0.957	0.148		0.768	0.797	0.825

*Non-RT/RT: the rectal cancer patients without/with preoperative radiotherapy. Median of relative expression with 25th−75th percentile is recorded in parentheses*.

**Table 4:** Correlation between miR-105, miR-302a, and miR-888 expression and biological factors in rectal patients without and with radiotherapy (RT).

	***n***	**Non-RT**			***n***	**RT**		
		**miR-105**	**miR-302a**	**miR-888**		**miR-105**	**miR-302a**	**miR-888**
**Apoptosis**								
<5%	22	0.028 (0.005–0.427)	0.571 (0.047–1.369)	2.935 (0.432–9.192)	17	0.058 (0.025–2.192)	0.194 (0.004–3.629)	0.036 (0.005–2.960)
≥5%	15	0.054 (0.014–1.260)	0.315 (0.104–3.710)	2.820 (0.287–118.657)	17	0.996 (0.012–3.195)	0.284 (0.081–2.121)	1.710 (0.321–16.405)
*P*-value		0.304	0.772	0.551		0.786	0.518	0.029
**Survivin**								
Negative	12	0.014 (0.006–0.888)	0.123 (0.044–1.430)	1.246 (0.078–2.635)	14	1.044 (0.016–4.760)	0.207 (0.021–0.766)	0.935 (0.143–4.673)
Positive	12	0.401 (0.021–5.089)	0.448 (0.083–0.774)	25.242 (2.726–108.535)	9	0.244 (0.068–3.885)	0.736 (0.054–9.295)	4.110 (0.070–22.050)
*P*-value		0.160	0.590	0.015		0.781	0.277	0.477
**Cox2**								
Weak	4	0.009 (0.002–0.014)	0.190 (0.051–3.903)	2.986 (0.149–25.543)	5	1.450 (0.163–5.675)	0.145 (0.014–3.629)	0.740 (0.016–42.200)
Strong	36	0.050 (0.012–1.240)	0.571 (0.080–3.070)	2.454 (0.099–16.875)	32	0.112 (0.016–3.693)	0.318 (0.020–1.083)	0.856 (0.055–5.308)
*P*-value		0.042	0.648	0.880		0.307	0.620	0.780
**p73**								
Negative	33	0.054 (0.013–1.220)	0.561 (0.059–2.584)	2.088 (0.057–20.225)	22	0.296 (0.035–6.095)	0.412 (0.008–8.453)	1.765 (0.030–7.125)
Positive	7	0.008 (0.001–0.041)	0.518 (0.065–3.606)	4.635 (1.788–7.993)	12	0.256 (0.011–2.613)	0.207 (0.066–0.738)	0.935 (0.183–5.085)
*P*-value		0.030	0.807	0.507		0.423	0.466	0.929
**PRL**								
Negative	5	0.006 (0.004–32.668)	0.128 (0.057–3.654)	1.182 (0.397–2.933)	4	7.525 (3.578–424.325)	0.741 (0.091–11.008)	3.410 (0.564–6.080)
Positive	34	0.045 (0.013–1.075)	0.540 (0.047–3.631)	2.454 (0.064–14.157)	30	0.071 (0.016–2.098)	0.252 (0.008–2.289)	0.661 (0.026–4.620)
*P*-value		0.192	0.855	0.294		0.013	0.519	0.392
**p130**								
Negative	33	0.041 (0.121–1.112)	0.561 (0.109–3.656)	2.090 (0.128–10.395)	24	0.096 (0.016–2.515)	0.122 (0.003–0.537)	0.661 (0.015–5.295)
Positive	6	0.237 (0.003–2.394)	0.314 (0.043–2.365)	28.180 (0.425–79.598)	8	0.236 (0.054–7.298)	6.370 (0.872–9.898)	5.438 (0.032–28.750)
*P*-value		0.924	0.556	0.349		0.254	0.002	0.428
**TEM1**								
Negative	23	0.047 (0.013–1.044)	0.561 (0.047–3.606)	3.779 (0.552–18.234)	21	1.820 (0.112–6.890)	0.402 (0.063–6.420)	4.110 (0.279–9.110)
Positive	16	0.024 (0.005–1.461)	0.405 (0.047–1.205)	0.988 (0.010–4.453)	15	0.018 (0.010–0.244)	0.194 (0.005–0.734)	0.184 (0.006–1.350)
*P*-value		0.373	0.832	0.121		0.003	0.238	0.025
**PPAR–δ**								
Weak	32	0.056 (0.011–1.695)	0.571 (0.066–3.808)	2.455 (0.099–11.600)	24	0.050 (0.011–0.334)	0.252 (0.005–0.821)	0.966 (0.015–6.080)
Strong	4	0.014 (0.007–0.035)	0.065 (0.001–0.633)	1.046 (0.001–59.173)	7	1.820 (0.116–5.300)	0.402 (0.100–10.500)	4.110 (0.112–10.500)
*P*-value		0.227	0.103	0.393		0.043	0.295	0.695
**WRAP53**								
Weak	13	0.014 (0.008–0.382)	0.047 (0.036–0.636)	1.790 (0.004–20.200)	21	0.116 (0.020–4.660)	0.734 (0.018–6.370)	1.130 (0.005–8.250)
Strong	23	0.042 (0.013–1.840)	0.801 (0.117–4.020)	2.090 (0.184–7.990)	10	0.112 (0.015–2.260)	0.202 (0.042–0.764)	1.171 (0.146–6.588)
*P*-value		0.434	0.015	0.379		0.663	0.268	0.441
**AEG-1**								
Weak	8	0.066 (0.009–0.968)	0.210 (0.117–0.680)	27.250 (4.250–346.550)	8	1.043 (0.109–9.480)	0.311 (0.185–2.758)	3.675 (0.419–9.805)
Strong	28	0.034 (0.011–1.695)	0.619 (0.470–3.918)	0.987 (0.010–5.225)	26	0.100 (0.016–3.578)	0.387 (0.007–6.350)	0.479 (0.011–5.820)
*P*-value		0.867	0.489	0.003		0.288	0.647	0.368
**SATB1**								
Weak	15	0.042 (0.006–1.260)	0.315 (0.104–3.840)	12.800 (2.090–78.200)	18	0.180 (0.029–4.340)	0.273 (0.004–6.350)	0.966 (0.023–6.053)
Strong	22	0.034 (0.013–0.357)	0.687 (0.038–3.635)	1.255 (0.008–5.478)	13	0.364 (0.029–7.525)	0.284 (0.031–1.036)	0.740 (0.022–3.050)
*P*-value		0.939	0.867	0.036		0.828	0.984	0.890

*Non-RT/RT: the rectal cancer patients without/with preoperative radiotherapy. Median of relative expression with 25th−75th percentile is recorded in parentheses*.

### miRNA Expression in Relation to the Patients With RT

In RT cases, the high expression of miR-105 was related to negative PRL-3 (*P* = 0.013), TEM1, *P* = 0.003), and strong PPAR-δ expression (*P* = 0.043). The high expression of miR-302a was associated with positive p130 (*P* = 0.002). The high expression of miR-888 was related to increased apoptosis (*P* = 0.029) and decreased TEM1 expression (*P* = 0.025, Table 4). No significant association was present between the expressions of miR-302a, miR-105, or miR-888 and clinicopathological features (*P* > 0.05, Table 3).

### miRNA Expression in Relation to Survival of the Patients

**Figure 2:**
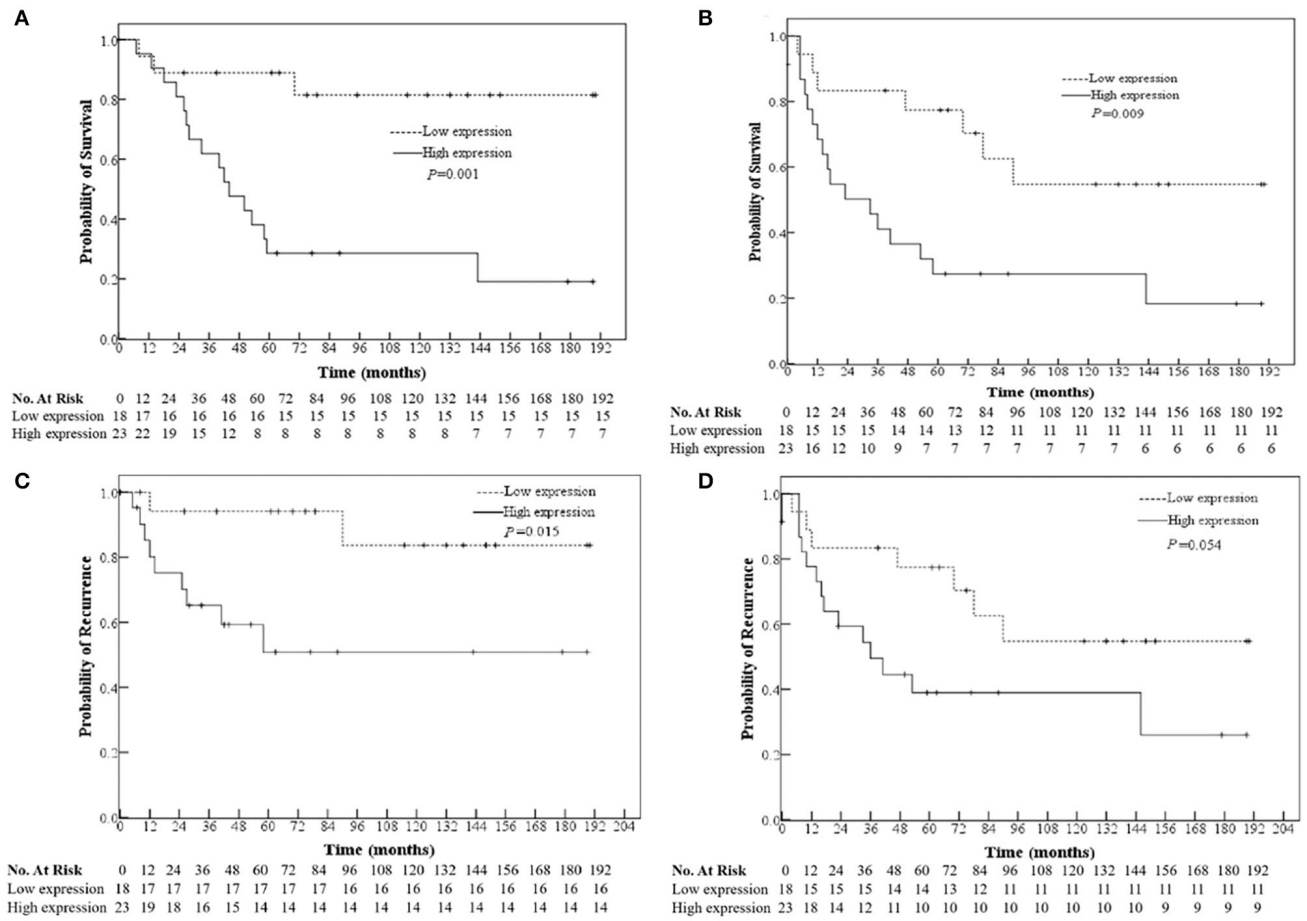
Relationships of the miR-888 expression in the primary rectal cancers with the patients' overall survival **(A)** and disease-free survival **(B)**, tumor location **(C)**, and distant recurrence **(D)** of non-RT patients.

RT has not shown any effect in conferring survival benefit for stage IV patients due to distant metastasis; thus, those patients are excluded from prognostic analysis In non-RT patients, univariate analysis showed that the high expression of miR-888 was correlated with worse overall survival (OS, *P* = 0.001), disease-free survival (DFS, *P* = 0.009), and local recurrence (*P* = 0.015) and had a trend relationship to distant recurrence (*P* = 0.054, Figure 2). Further, a multivariate analysis revealed the prognostic significance of OS and DFS remained, independent of gender, age, TNM stage, and differentiation [OS: hazard ratio (HR), 4.881; *P* = 0.015; 95% confidence interval (CI), 1.369–17.406; DFS: HR, 2.627; *P* = 0.039; 95% CI, 1.052–6.560; Table 5]. There were no significant relationships between the expressions of miR-105 or miR-302a and survival regardless of RT (*P* > 0.05).

**Table 5:** Multivariate analysis of miR-888 expression with overall survival and disease-free survival in rectal cancer patients without radiotherapy (RT).

**Characteristics**	**OS**		**DFS**	
	**RR (95%CI)**	***P*-value**	**RR (95%CI)**	***P*-value**
**miR-888 expression**				
Low vs. High	6.134 (1.754–21.459)	0.005	3.071 (1.246–7.568)	0.015
**Gender**				
Male vs. Female	0.873 (0.320–2.380)	0.790	0.587 (0.240–1.434)	0.242
**Age (year)**				
<70 vs. ≥70	0.934 (0.374–2.334)	0.884	0.933 (0.410–2.123)	0.869
**Histological grade**				
Well + moderate vs. Poor + mucinous	0.725 (0.226–2.331)	0.590	0.863 (0.296–2.513)	0.787
**TNM stage**				
I *vs*. II + III + IV	5.791 (1.317–25.468)	0.020	2.703 (0.995–7.348)	0.051

*OS, overall survival; DFS, disease-free survival; RR, relative risk*.

### Biological Functional Analysis of miR-302a, miR-105, and miR-888

**Figure 3:**
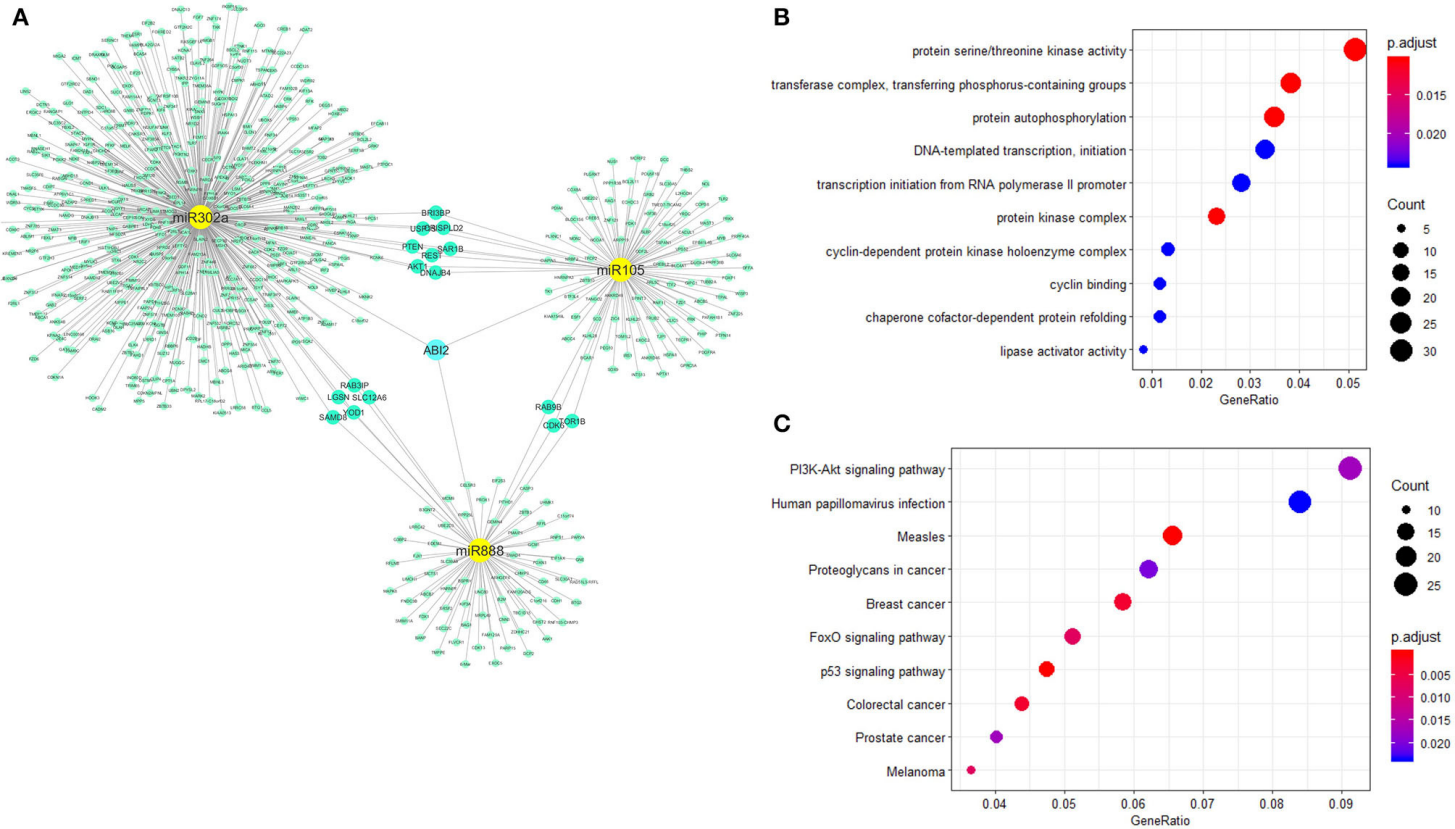
Biological functional analysis of miR-302a, miR-105, and miR-888. The relationships of miR888, miR105, and miR302a were analyzed with the miRNA–gene interaction network. ABI2 revealed as the key interaction molecule for these three miRNAs **(A)**. Protein serine/threonine kinase activity was the most significantly enriched function for these miRNAs-related genes from GO annotation **(B)**. PI3K-Akt signaling pathway showed high correlation with these miRNAs-related genes from KEGG **(C)**.

miRNet was used for network creation and analysis. The miRNA–gene network for these three miRNAs was shown, in which several genes had relationships with more than one miRNA (Figure 3A). GO annotation showed that protein serine/threonine kinase activity was the most significantly enriched functions for these miRNA-related genes (Figure 3B). PI3K-AKT signaling pathway showed high correlation with these miRNA-related genes from KEGG pathway analysis (Figure 3C).

**Figure 4:**
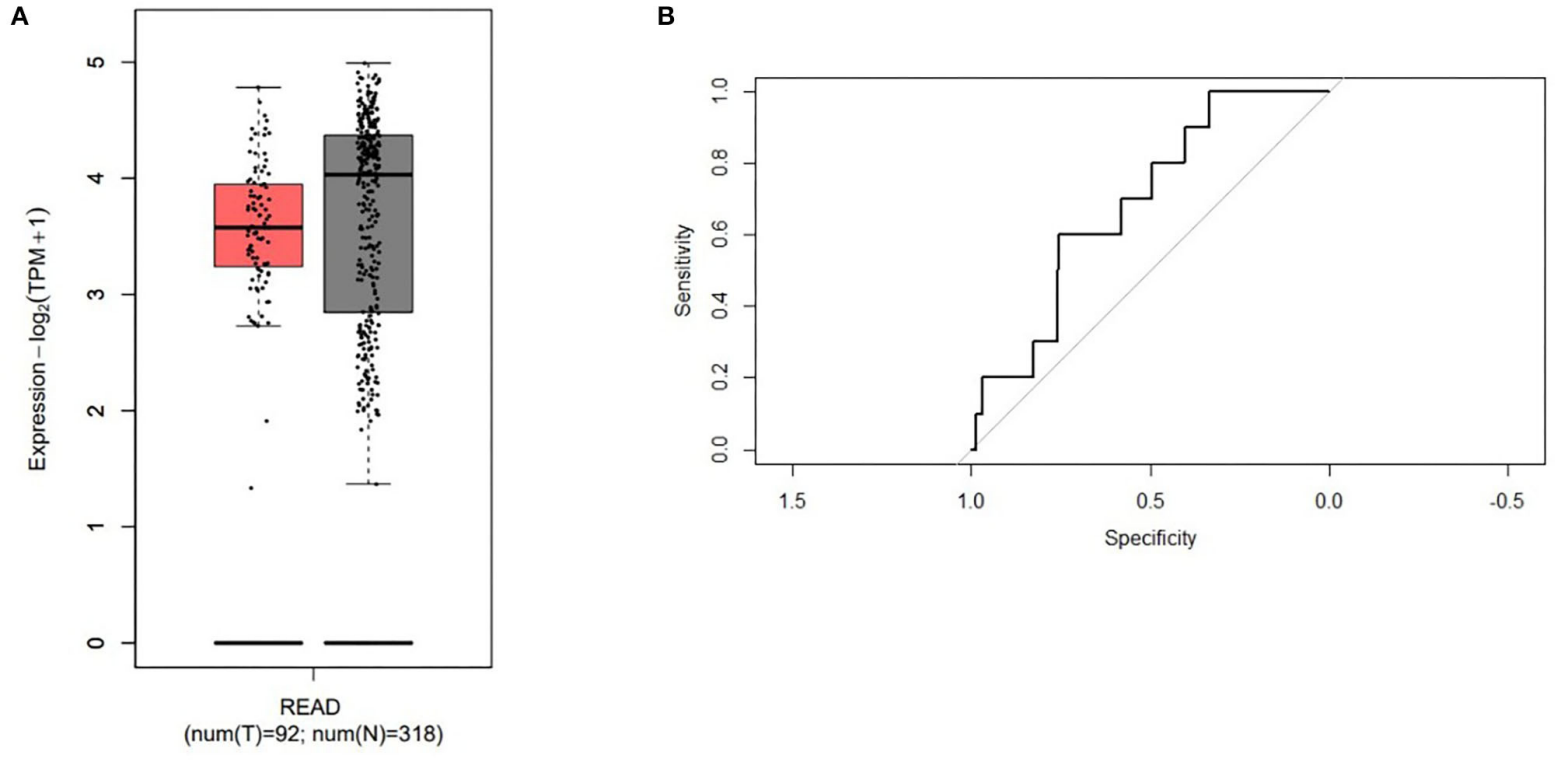
The different expression of the ABI2 in the rectal cancer (red box) and the normal controls (gray box) **(A)** and the diagnostic values **(B)** were represented.

**Figure 5:**
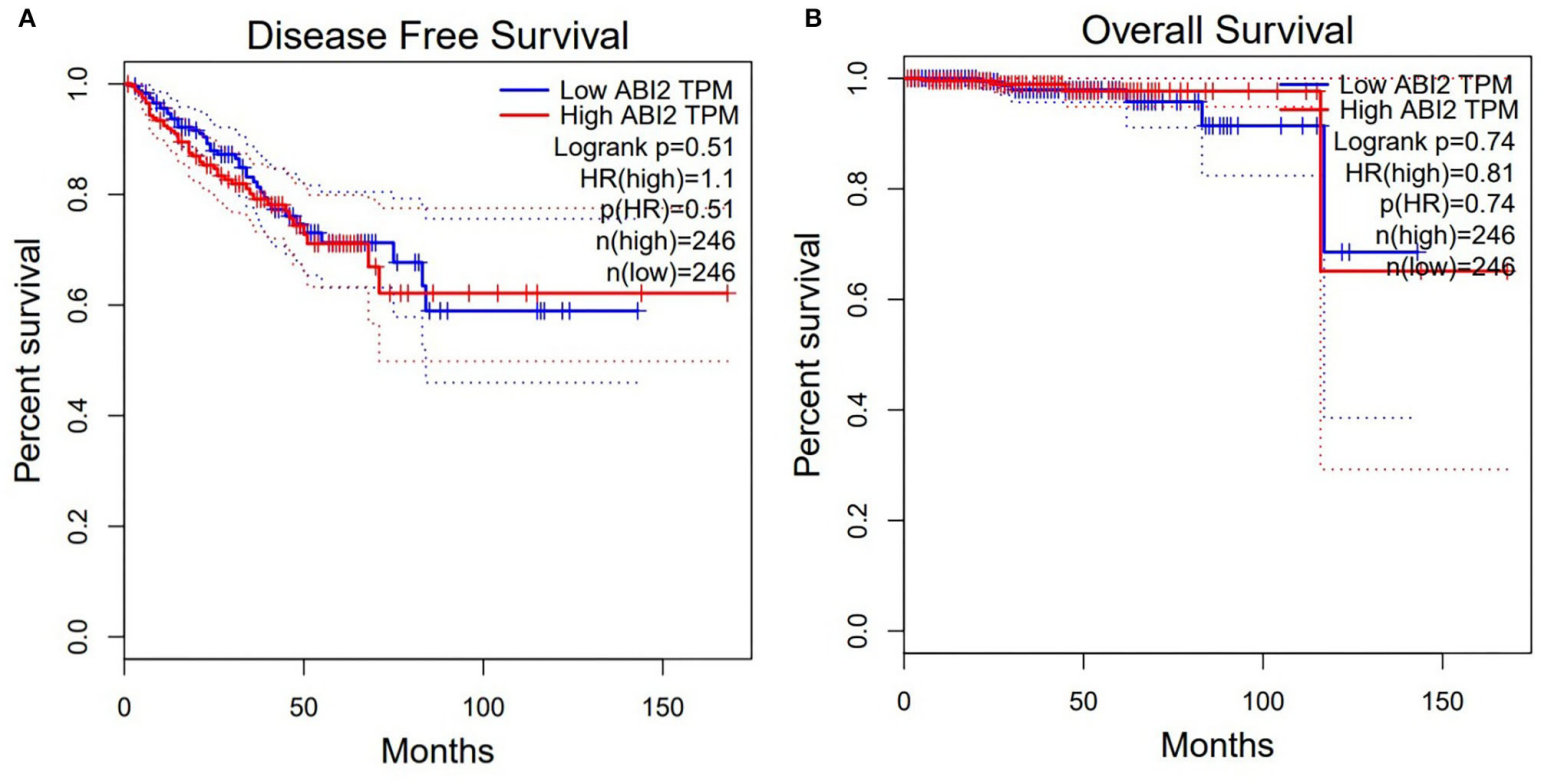
Survival analysis showed that the ABI2 expression was not associated with disease-free survival **(A)** and overall survival **(B)** in rectal cancer patients.

Moreover, the expression of ABI2, which was associated with all the three miRNAs, was compared between rectal cancers (*n* = 92) and normal controls (n = 318) based on the GEPIA database. ABI2 expression in cancers was significantly lower than that in normal controls (Figure 4A). Further, the diagnostic values of ABI2 expression were analyzed in 167 rectal cancer patients and 10 normal controls based on the TCGA database. Wilcoxon test showed that these two groups had a significant difference (*P* = 0.046), with an area under the curve (AUC) of 0.688 for diagnostic ROC curve (Figure 4B). Finally, the prognostic value of ABI2 expression was determined by survival analysis. The results showed that ABI2 expression was not associated with the DFS or OS in rectal cancer patients (Figure 5). Taken together, these results indicate the oncogenic feature of these three miRNAs in pathogenesis of rectal cancer based on ABI2 expression.

### Analysis of miR-302a, miR-105, and miR-888 Expression in TCGA Database

To validate the role of miR-302a, miR-105, and miR-888 expression in rectal cancer, we used the TCGA database and obtained the expression for a panel of rectal cancers (*n* = 159). The range of miR-105 in rectal cancer tissue was 0–1,442, mean value with standard deviation was 80.12 ± 249.29. Mean values with standard deviation of miR-302a and miR-888 in rectal cancers were 0.04 ± 0.22 (0–2) and 0.06 ± 0.30 (0–3), respectively. The expressions of the three miRNAs in rectal cancers were not different from those in normal rectal tissue *(P* > 0.05). Furthermore, the patients were also classified into a low expression group and a high expression group based on the median value (1) of miR-105 expression, along with the median value (0) of miR-302a and miR-888 expression.

Moreover, we investigated the expression of these miRNAs with clinicopathological features in 159 rectal cancers. Using the TCGA database, we did not see the significant association of miR-302a, miR-105, or miR-888 expression with clinicopathological features (*P* > 0.05, Table 6), which was almost in accordance with our results above. Nevertheless, we found that the expression of miR-105 was significantly higher in rectal cancers with high carcinoembryonic antigen (CEA) level (*P* = 0.048). Ultimately, we analyzed the association between these miRNAs expression in rectal cancer and prognosis based on the data retrieved by TCGA. There was no significant relationship between the expression of miR-302a, miR-105, or miR-888 and OS (*P* > 0.05).

**Table 6:** Correlation of clinicopathological characteristics and miR-302a, miR-105, and miR-888 expression in the rectal cancer patients based on TCGA.

	***n***	**miR-105 expression**		**miR-302a expression**		**miR-888 expression**	
		**High**	**Low**	**High**	**Low**	**High**	**Low**
**Tumor vs. mucosa**							
Rectal tumor	159	78 (49.1)	81 (50.9)	6 (3.8)	153 (96.2)	7 (4.4)	152 (95.6)
Normal mucosa	3	3 (100)	0 (0)	0 (0)	3 (100)	0 (0)	3 (100)
*P*-value		0.24	0.23	0.29			
**Gender**							
Male	86	36 (41.9)	50 (58.1)	3 (3.5)	83 (96.5)	4 (4.6)	83 (95.4)
Female	73	42 (57.5)	31 (42.5)	2 (2.7)	71 (97.3)	3 (5.5)	69 (94.5)
*P*-value		0.07	0.86	0.79			
**Age (year)**							
≤66	87	41 (47.1)	46 (52.9)	4 (4.6)	83 (95.4)	2 (2.3)	85 (97.7)
>66	72	37 (51.4)	35 (48.6)	1 (1.4)	71 (98.6)	5 (6.9)	67 (93.1)
*P*-value		0.71	0.48	0.30			
**TNM stage**							
I	29	14 (48.3)	15 (51.7)	0 (0)	29 (100)	2 (6.9)	27 (93.1)
II	47	23 (48.9)	24 (51.1)	1 (2.1)	46 (97.9)	1 (2.1)	46 (97.9)
III	50	21 (42)	29 (58)	4 (8)	46 (92)	2 (4)	48 (96)
IV	24	14 (58.3)	10 (41.7)	0 (0)	24 (100)	1 (4.2)	23 (95.8)
NA	9	6 (66.7)	3 (33.3)	0 (0)	9 (100)	1 (11.1)	8 (88.9)
*P*-value		0.566	0.196	0.738			
**Tumor histology**							
Adenocarcinoma	140	71 (50.7)	69 (49.3)	5 (3.6)	135 (96.4)	6 (4.3)	134 (95.7)
Mucinous	14	4 (28.6)	10 (71.4)	0 (0)	14 (100)	0 (0)	14 (100)
NA	5	3 (60)	2 (40)	0 (0)	5 (100)	1 (20)	4 (80)
*P*-value		0.254	0.705		0.170		
**Lymphatic invasion**							
No	78	39 (50)	39 (50)	3 (3.8)	75 (96.2)	3 (3.8)	75 (96.2)
Yes	63	30 (47.6)	33 (52.4)	2 (3.2)	61 (96.8)	4 (6.3)	59 (93.7)
NA	18	9 (50)	9 (50)	0 (0)	18 (100)	0 (0)	18 (100)
*P*-value		0.956	0.701	0.484			
**Venous invasion**							
No	103	50 (48.5)	53 (51.5)	3 (2.9)	100 (97.1)	5 (4.9)	98 (95.1)
Yes	34	17 (50)	17 (50)	2 (5.9)	32 (94.1)	2 (5.9)	32 (94.1)
NA	22	11 (50)	11 (50)	0 (0)	22 (100)	0 (0)	22 (100)
*P*-value		0.985	0.456	0.538			
**Preoperative CEA**							
Mean ± SD	106	228.88 ± 173.91	66.26 ± 340.83	5.97 ± 3.60	150.53 ± 866.88	2.92 ± 3.10	150.67 ± 866.85
*P*-value		0.048	0.711	0.705			

*NA, not available*.

## Discussion

miRNAs are emerging as major regulatory factors in normal and tumor cell processes and have been demonstrated to be involved in oncogenesis, tumor progression, invasion, and metastasis (44). To identify potential miRNAs in relation to other biomarkers, RT response, and clinical outcome in rectal cancers, we examined the relative levels of miR-302a, miR-105, and miR-888 in cancer and normal samples from a cohort of 80 rectal cancer patients with a Swedish clinical trial of preoperative RT. In non-RT cases, the expression of miR-105 in rectal cancers was significantly higher than that in normal rectal samples. Moreover, the expression of miR-105 was significantly higher in rectal cancers with mucinous histological type, more frequently in cancers with strong COX-2 and p73 expression. Previous studies showed that the expressions of p73 and COX-2 were significantly increased in primary rectal cancers compared with normal mucosa samples, indicating that both of them are involved in rectal cancer development (32, 41). We found that the expression of miR-105 was significantly higher in rectal cancers with high CEA level based on the TCGA data. CEA is a complex glycoprotein produced by 90% of CRCs and modulates intercellular adhesion and functions as a promoter of cellular aggregation (45). A recent study demonstrated the modulatory role of miR-105 in tumor necrosis factor α-induced epithelial–mesenchymal transition and further CRC metastasis (10). These findings suggested an aggressive cancer phenotype of miR-105, which might be important for rectal cancer development and progression. We further examined the expression of miR-105 in patients undergoing RT and found that miR-105 expression was related to negative PRL-3 and TEM1 expression and positive PPAR-δ expression. Our previous studies showed that the weak expression of PRL-3 and strong expression of PPAR-δ predicted favorable survival in rectal cancer patients with preoperative RT (35, 38). Together with these findings, our results suggest the involvement of miR-105 in pathogenesis and imply its potential role in radiation response for rectal cancer patients.

In the present study, there was no difference of either miR-302a or miR-888 expression between rectal cancers and normal rectal samples without RT, which was consistent with the results based on the TCGA-retrieved data. In contrast, the previous study by Bobowicz et al. (20) revealed that miR-302a and miR-888 were overexpressed in malignant tissue as compared with normal mucosa. The objects of this study were restricted to T_2−3_N_0_ colon cancer patients; this might contribute to the variations seen in our results.

Moreover, our results showed that the expression of miR-888 was significantly higher in cancers with strong survivin and weak AEG-1 and SATB1 expression. In fact, miR-888 is upregulated in some urogenital malignant tumors and plays a potential role in cancer progression. Upregulated miR-888 expression was suggested to have an important function in development and progression of endometrial tumors (16, 17). Likewise, a recent study showed that the overexpression of miR-888 increased cellular proliferation and migration by targeting the tumor suppressor genes RBL1 and SMAD4, suggesting the oncogenic role of miR-888 in prostate cancer progression (13). The latest study validated RBL1, KLF5, SMAD4, and TIMP2 as the direct miR-888 targets in prostate cancer (14). MiR-888 upregulated in MCF-7 side population cells by targeting E-Cadherin expression, which indicates its potential role in metastasis of breast cancer (18). In HCC, miR-888 increased the expression of MMP-2 and MMP-9 proteins, which contribute to cell migration and invasion; miR-888 also decreased the expression of p53 protein, which further promoted malignancy (19). Indeed, the target prediction by TargetScan, a superior program with the best performance in comparison, also identified AEG-1 and SATB1 as putative targets for miR-888 in the present study. Our previous studies had identified the role of SATB1 and AEG-1 in the progression of rectal cancer, as well as the involvement of the nuclear factor κB signaling pathway for AEG-1 and potential interaction in response to radiation through different canonical pathways for SATB1 by Western blotting and qRT-PCR analysis (40, 42, 46). Thus, these findings are consistent with the results of our present study, which points to an oncogenic role for miR-888 in rectal cancer by modulating targets such as AEG-1 and SATB1. These results may suggest that blocking miR-888 could be an effective therapeutic approach in rectal cancer patients.

Importantly, the present study showed that the high expression of miR-888 was independently related to worse survival and had a potential relationship to recurrence in primary rectal cancer without RT. Moreover, high miR-888 expression was related to heavily increased apoptosis and less TEM1 expression in RT patients, and RT may reduce the expression of miR-888. Previous studies showed that TEM1 expression in tumor cells was positively related to PRL expression, whereas weak PRL-3 expression predicts favorable survival in rectal cancer patients with preoperative RT (35, 36). Therefore, these results suggest that miR-888 can serve as a potential biomarker for preoperative RT and clinical outcome in rectal cancer. The survival benefit from miR-888 expression was not observed in the TCGA database, which was probably attributed to the few samples of normal rectal mucosa in the TCGA database.

Although we could not find a difference of miR-302a expression between rectal cancers and normal rectal samples in non-RT patients, the expression of miR-302a was significantly higher in rectal cancers with necrosis and strong WRAP53 expression. However, when the predicted targets identified by TargetScan were compared with our experimental results, we found there was no overlap for miR-302a. Most likely being that the miRNA targets were tissue-specific. This result illustrated the well-documented difficulties of predicting targets for miRNA (47). Our previous study showed that tumor necrosis was related to advanced stage and worse survival in rectal cancer patients (48), and WRAP53 level was significantly increased in primary tumor compared to normal mucosa, which was associated with poor prognosis (39). In fact, the reduced expression of miR-302a has been frequently reported in several types of malignancies, such as breast cancer (21), HCC (22), prostate cancer (23), cervical cancer (24), and skin cancer (25, 26). However, miR-302a was found to be overexpressed in CRC (20, 28, 29). MiR-302a overexpression inhibits proliferation and invasion of CRC cells by reducing the expression of related proteins through suppression of MAPK and PI3K/AKT signaling pathways (28, 29). Taken together, these studies suggest the distinct role of miR-302a expression in the pathogenesis of CRC, and further studies are needed to determine its role in CRC.

The miRNA–gene network analysis showed that several genes had relationships with more than one miRNA. Biological functional analysis revealed the protein serine/threonine kinase activity and PI3K-AKT signaling pathway were the most significantly enriched function and pathway, respectively. Furthermore, the expression of ABI2, a target related with all three miRNAs, was significantly lower in rectal cancer tissues than that in normal controls, with an AUC of 0.688 for diagnostic ROC curve. As we know, ABI-2, a member of the ABI family of adaptor proteins, has been proven to be involved in signaling pathways involving tyrosine kinase and Rac GTPase. A previous study showed that ABI2 functions as a tumor suppressor and a cell migration inhibitor (49). Thus, these results indicate the oncogenic feature of the three miRNAs in pathogenesis of rectal cancer based on the data of ABI2 expression.

In RT patients, the high expression of miR-302a was related to positive p130, and RT might decrease the expression of miR-302a. A recent study showed that miR-302a sensitized breast cancer cells against RT. In addition, the expression of miR-302a was inversely correlated with those of AKT1 and RAD52, the two critical regulators of radioresistance (30). Together with these findings, our study provides new evidence on RT response in rectal cancers and suggests that miR-302a may be a potential sensitizer for RT therapy in rectal cancers.

Biological functional analysis in the current study showed that protein serine/threonine kinase activity was the most significantly enriched function for these miRNA-related genes. The serine–threonine kinases are an important family of proteins that modulate the phosphorylation of many key effectors of the apoptotic process, which includes key mediators of carcinogenesis such as RAF, AKT/protein kinase B, and MEK. The enriched functions of protein kinases for these miRNAs suggested their key roles in CRC carcinogenesis. Likewise, PI3K/AKT signaling pathway showed high correlation with these miRNA-related genes from KEGG. It is well-known that the PI3K/AKT signaling pathway plays a vital role in cell survival. Aberrant activation of this signaling cascade is associated with CRC development and progression (50). In addition, the prosurvival function of PI3K/AKT signaling is expected to positively contribute to the radioresistance of cancer cells. The inhibition of PI3K/AKT signaling leads to an enhancement of radiosensitivity of cancer cells both *in vitro* and *in vivo*. Furthermore, the increase in radiosensitivity by PI3K/AKT inhibition involves both the diminution of DNA repair and an enhancement of apoptosis induction. Therefore, regulatory oncogenic or tumor suppressor miRNAs for PI3K/AKT signaling regulate cellular proliferation, migration, invasion, angiogenesis, and resistance to chemotherapy/RT in CRC (51).

In the future, we will work on the limitations of our present study. We have planned to collect more rectal cancer patients with the RT to confirm the present results and further to examine these miRNA effects on rectal cancer patients with different RT strategies, such as varying doses and fractions. Besides, the deeper biological investigation is needed to identify the functional roles and signaling pathways of these miRNAs.

This study showed that expression of miR-105 was upregulated in the rectal cancers as compared with the normal samples regardless of RT, and the high expression of miR-105 and miR-888 was associated with aggressive clinicopathological features. TargetScan predicted AEG-1 and SATB1 as putative targets for the miR-888. The TCGA database demonstrated that expression of the miR-105 was associated with high level of CEA. Moreover, RT decreased the expressions of the miR-105, miR-302a, and miR-888. More importantly, the increased expression of miR-888 was independently related to the worse prognosis in the rectal cancer patients without RT. Our findings suggested that the miR-105 was involved in pathogenesis and miR-888 in prognosis. The expression of miR-302a, miR-105, and miR-888 played potential roles in RT response for rectal cancer patients.

## Clinical Perspectives

- The overexpression of miR-105 in the rectal cancers as compared with the normal rectal tissue may provide further evidence to confirm the diagnosis for rectal cancer.
- The overexpression of miR-105 and miR-888 may be indicators for aggressive clinicopathological features of rectal cancers.
- The decreased expression of miR-302a, miR-105, and miR-888 may play potential roles in RT response for rectal cancer patients.
- The overexpression of miR-888 was associated with worse outcome in rectal cancer patients.

## Data Availability Statement

All datasets presented in this study are included in the article/Supplementary Material.

## Author Contributions

X-FS designed the study. BH prepared tissue arrays. W-JM, HZ, and X-FS performed immunohistochemical studied. IJ and GA collected clinical data. XZ and HZ analyzed networks. W-JM and SP collected and analyzed the data. W-JM, AZ, HZ, and X-FS wrote and revised the manuscript. Z-QW, HZ, Z-GZ, and X-FS coordinated the study. All authors read and approved the manuscript.

## Conflict of Interest

The authors declare that the research was conducted in the absence of any commercial or financial relationships that could be construed as a potential conflict of interest.

## Supplementary Material

Supplementary 1 Reverse transcription and qRT-PCR; Normalization and data analysis; Immunohistochemistry; TUNEL assay.

Supplementary 2 The predicted targets of miR-302a, miR-105, and miR-888 by TargetScan.

## Abbreviations

AEG-1: astrocyte elevated gene-1
AKT: a serine/threonine-specific protein kinase
CEA: carcinoembryonic antigen
CRC: colorectal cancer
GEPIA: gene expression profiling interactive analysis
DFS: disease-free survival
GO: gene ontology
HCC: hepatocellular carcinoma
HE: hematoxylin and eosin
KEGG: Kyoto encyclopedia of genes and genomes
MAPKs: mitogen-activated protein kinases
MEK: mitogen-activated protein kinase
miRNAs: micoRNAs
OS: overall survival
PRL-3: phosphatase of regenerating liver-3
RT: radiotherapy
SATB1: special AT-rich sequence binding protein 1
TCGA: the cancer genome atlas
TNM: classification of malignant tumors
TUNEL: transferase-mediated deoxyuridine riphosphate-biotin nick end labeling assay.
