# Supplementary material for: Expressions of miR-302a, miR-105, and miR-888 Play Critical Roles in Pathogenesis, Radiotherapy, and Prognosis on Rectal Cancer Patients: A Study From Rectal Cancer Patients in a Swedish Rectal Cancer Trial of Preoperative Radiotherapy to Big Database Analyses
Source: Front Oncol. 2020 Oct 6;10:567042. doi: 10.3389/fonc.2020.567042 (PMC7573294; doi:10.3389/fonc.2020.567042)
Supplement: Supplementary 1 — Reverse transcription and qRT-PCR; Normalization and data analysis; Immunohistochemistry; TUNEL assay. [file Data_Sheet_1.doc]

**Supplementary 1**

*Reverse transcription and qRT-PCR*

cDNA was synthesized using gene-specific primers according to the TaqMan MicroRNA Assay protocol (Applied Biosystems). For reverse transcription, 10 ng of RNA sample, 0.25 mM of each dNTPs, 3.33 U/μl of MultiScribe reverse transcriptase, 50 nM of stem-loop RT primer, 1× RT buffer and 0.25 U/μl of RNase inhibitor (all from TaqMan MicroRNA Reverse Transcription kit; Applied Biosystems) were used. Reaction mixtures were incubated for 30 min. at 16°C, 30 min. at 42°C, 5 min. at 85°C and then held at 4°C (TGradient thermal cycler; Biotherma). Real-time PCR was performed using the Applied Biosystems 7900HT Fast Real-Time PCR System. The 20 μl PCR reaction mixtures consisted of 1.33 μl of RT product, 1× TaqMan (NoUmpErase UNG) Universal PCR Master Mix and 1 μl of primer and probe mix of the TaqMan MicroRNA Assay kit (Applied Biosystems). Reactions were run in a 96-well optical plate at 95°C for 10 min., followed by 40 cycles at 95°C for 15 sec. and 60°C for 10 min. Comparative real-time PCR was performed in duplicate and included no-template controls.

*Normalization and data analysis*

The CT values (CT) were calculated by SDS 2.4.1 software (Applied Biosystems) using the manual threshold settings (threshold=0.2). RNU6b (Assay No. 001006; Applied Biosystems) was selected as reference gene. Relative expression level in cancer and normal samples was analyzed by the ΔCt method (ΔCt= Ct of miRNA of interest- Ct of RNU6b). Besides, the fold change in miRNA expression was calculated, with the mean Ct among all normal samples being used as the calibrator, by using the 2^-ΔΔCt^ method [44]. Thus, miRNA expressions were dichotomized in survival analysis, with fold change more than one as high expression while less than one as low expression.

*Immunohistochemistry*

Immunohistochemistry was performed at our laboratory for the following biomarkers: p73 (*n*=74) [32], p130 (*n*=71) [45], PRL-3 (*n*=73) [35], TEM1 (*n*=75) [37], survivin (*n*=47) [46], PPAR-δ (*n*=67) [36], Wrap53 (*n*=67) [40], AEG-1 (*n*=70) [47], COX-2 (*n*=77) [33] and SATB1 (*n*=68) [48]. Tissue sections were incubated at 60 °C for overnight, dewaxing and hydrated. The activity of endogenous peroxidase was blocked in 3% H_2_O_2_ in methanol. Nonspecific background staining was immersed in PBS containing 1% BSA and incubated with protein block solution (Spring Bioscience, Pleasanton, CA) for 10 min. The sections were then incubated with individual primary antibody and the corresponding secondary antibody. The sections were then rinsed in PBS and incubated for 10 min in peroxidase substrate containing 3, 3-diaminobenzidine (DAB) chromogen and counterstained with hematoxylin. In all runs, negative and positive controls were included.

All immunohistochemical sections for each biomarker were independently reviewed scored by two investigators (including one pathologist) without knowledge of clinicopathological and biological information. In the case of discrepancy in individual scores, both investigators re-evaluated the sections together and reached a consensus before combining the individual scores. To avoid an artificial effect, the cells on the margins of the sections and in areas with poor morphology were not counted.

*TUNEL assay*

Apoptotic cells were detected by the terminal deoxynucleotidyl transferase-mediated dUTP-biotin nick end-labelling (TUNEL) assay [49]. The 5 μm-thick sections were cut from the paraffin blocks of the surgical specimen. The sections were dewaxed in xylene, rehydrated, and incubated with 20 μg/mL proteinase K (Boehringer-Mannheim Biochemicals, Indianapolis, IN) for 15 min and rinsed in distilled water. Endogenous peroxidase activity was inhibited with 2% hydrogen peroxide. The ApopTag® In Situ Apoptosis Detection Kit (Oncor Inc., Gaithersburg, MD) was used to detected apoptosis. The sections were then incubated with equilibration buffer for 10-15 sec and TdT enzyme in humidified atmosphere at 37 ^◦^C for 90 min. They were subsequently put into prewarmed working strength stop/wash buffer at room temperature for 10 min and incubated with antidigoxigenin-peroxidase for 45 min. Staining was performed with 0.05% 3, 3-diaminobenzidine tetrahydrochloride (Sigma Chemical Co. St. Louis, MO), and counter staining was performed in methyl green. A section from rat mammary gland (Oncor Inc.) was included in each run as a positive control. To produce DNA fragments, the control sections were treated with 2 μg/mL DNAase at 37 ^◦^C for 30 min before the control sections by TUNEL assay were labeled.
